# Empirical Lossless Compression Bound of a Data Sequence

**DOI:** 10.3390/e27080864

**Published:** 2025-08-14

**Authors:** Lei M. Li

**Affiliations:** 1 State Key Laboratory of Mathematical Science, Academy of Mathematics and Systems Science, Chinese Academy of Sciences, Beijing 100190, China; lilei@amss.ac.cn; 2 School of mathematical sciences, University of Chinese Academy of Sciences, Beijing 100049, China

**Keywords:** lossless compression, entropy, normalized maximum likelihood, local asymptotic normality, predictive, Bayesian, DNA

## Abstract

We consider the lossless compression bound of any individual data sequence. Conceptually, its Kolmogorov complexity is such a bound yet uncomputable. According to Shannon’s source coding theorem, the average compression bound is nH, where *n* is the number of words and *H* is the entropy of an oracle probability distribution characterizing the data source. The quantity $nH({\hat{\theta }}_{n})$ obtained by plugging in the maximum likelihood estimate is an underestimate of the bound. Shtarkov showed that the normalized maximum likelihood (NML) distribution is optimal in a minimax sense for any parametric family. Fitting a data sequence—without any *a priori* distributional assumption—by a relevant exponential family, we apply the local asymptotic normality to show that the NML code length is $nH\left({\hat{\theta }}_{n}\right)+\frac{d}{2}\log\;\frac{n}{2\pi }+\log\int_{\Theta }{\left|I\left(\theta \right)\right|}^{1/2}\;d\theta +o\left(1\right)$, where *d* is dictionary size, |I(θ)| is the determinant of the Fisher information matrix, and Θ is the parameter space. We demonstrate that sequentially predicting the optimal code length for the next word via a Bayesian mechanism leads to the mixture code whose length is given by $nH\left({\hat{\theta }}_{n}\right)+\frac{d}{2}\log\;\frac{n}{2\pi }+\log\frac{|\;I\left({\hat{\theta }}_{n}\right){|}^{1/2}}{w({\hat{\theta }}_{n})}+o\left(1\right)$, where w(θ) is a prior. The asymptotics apply to not only discrete symbols but also continuous data if the code length for the former is replaced by the description length for the latter. The analytical result is exemplified by calculating compression bounds of protein-encoding DNA sequences under different parsing models. Typically, compression is maximized when parsing aligns with amino acid codons, while pseudo-random sequences remain incompressible, as predicted by Kolmogorov complexity. Notably, the empirical bound becomes more accurate as the dictionary size increases.

## 1. Introduction

The computation of the compression bound of any individual sequence is both a philosophical and a practical problem. It touches on the fundamentals of human intelligence. After several decades of effort, many insights have been gained by experts from various disciplines.

In essence, the bound is the length of the shortest program that prints the sequence on a Turing machine, referred to as the Solomonoff–Kolmogorov–Chaitin algorithmic complexity. Under this framework, if a sequence cannot be compressed by any computer program, it is considered random. On the other hand, if we can compress the sequence using a certain program or coding scheme, then it is not random, and we uncover some pattern or knowledge within the sequence. Nevertheless, Kolmogorov complexity is not computable.

Along another line, the source coding theorem proposed by Shannon [1] claimed that the average shortest code length is no less than nH, where *n* is the number of words and *H* is the the entropy of the source, assuming its distribution can be specified. Although Shannon’s probabilistic framework has inspired the inventions of some ingenious compression methods, nH is an oracle bound. Some further questions need to be addressed. First, where does the probability distribution come from? A straightforward approach is to infer it from the data themselves. However, in the case of discrete symbols, plugging in the empirical word frequencies ${\hat{\theta }}_{n}$ observed in the sequence results in $nH({\hat{\theta }}_{n})$, which can be shown to be an underestimate of the bound. Second, the word frequencies are counted according to a dictionary. Different dictionaries yield different distributions and thus different codes. What is the criterion for selecting a good dictionary? Third, the behavior of some compression algorithms, such as Lempel–Ziv coding, shows that as the sequence length increases, the size of the dictionary also grows. What is the exact impact of the dictionary size on the compression? Fourth, can we achieve the compression limit using a predictive code that processes the data in only one pass? Fifth, how is the bound derived from the probabilistic framework, if at all, connected to the conclusions drawn from the algorithmic complexity?

In this article, we review the key ideas of lossless compression and present some new mathematical results relevant to the aforementioned problems. Besides the algorithm complexity and the Shannon source coding theorem, the technical tools center around the normalized maximum likelihood (NML) coding [2,3] and predictive coding [4,5]. The expansions of these code lengths lead to an empirical compression bound that is indeed sequence specific and naturally linked to algorithmic complexity. Although the primary theme is the pathwise asymptotics, their related average results are also discussed for the sake of comparison. The analytical results apply not only to discrete symbols but also to continuous data provided the codelength for the former is replaced by the description length for the latter [6]. Other than theoretical justification, the empirical bound is exemplified by protein-coding DNA sequences and pseudo-random sequences.

## 2. A Brief Review of the Key Concepts

### 2.1. Data Compression

The basic concepts of lossless coding can be found in the textbook [7]. Before we proceed, it is helpful to clarify the jargon used in this paper: strings, symbols, and words. We illustrate them with an example. The following “studydnasequencefromthedatacompressionpointofviewforexampleabcdefghijklmnopqrstuvwxyz”, is a string. The 26 small case distinct English letters appearing in the string are called symbols, and they form an alphabet. If we parse the string into “study”, “dnasequence”, “fromthedata”, “compressionpointofview”, “forexample”, “abcdefg”, “hijklmnopq”, and “rstuvwxyz”, these substrings are called words.

The implementation of data compression involves an encoder and a decoder. The encoder parses the string to be compressed into words and replaces each word by its codeword. This produces a new string, which is hopefully shorter than the original one in terms of bits. The decoder, conversely, parses the new string into codewords, and interprets each codeword back to a word of the original symbols. The collection of all distinct words in a parsing is called a dictionary.

In the context of data compression, two issues arise naturally. First, is there a lower bound? Second, how do we compute this bound, or is it computable at all?

### 2.2. Prefix Code

A fundamental concept in lossless compression is the prefix code or instantaneous code. A code is called a prefix one if no codeword is a prefix of any other codeword. The prefix constraint has a close relationship to the metaphor of the Turing machine, by which the algorithmic complexity is defined. Given a prefix code over an alphabet of α symbols, the codeword lengths $l_{1}$, $l_{2}$, ⋯, $l_{m}$, where *m* is the dictionary size, must satisfy the Kraft inequality: $\sum_{i=1}^{m}\;\alpha^{-l_{i}}\leq 1$. Conversely, given a set of code lengths that satisfy this inequality, there exists a prefix code with those code lengths. Note that the dictionary size in a prefix code could be either finite or countably infinite.

The class of prefix codes is a subset of the more general class of uniquely decodable codes, and one may expect that some uniquely decodable codes could be advantageous over prefix codes in terms of data compression. However, this is not necessarily the case, for it can be shown that the codeword lengths of any uniquely decodable code must satisfy the Kraft inequality. Therefore, a prefix code can always be constructed to match the codeword lengths of any given uniquely decodable code.

A prefix code has an attractive self-punctuating feature: it can be decoded without reference to the future codewords since the end of a codeword is immediately recognizable. For these reasons, prefix coding is commonly used in practice. A conceptual yet convenient generalization of the Kraft inequality is to drop the integer requirement for code lengths and ignore the effect of rounding. A general set of code lengths can be implemented by the arithmetic coding [8,9]. This generalization leads to a correspondence between probability distributions and prefix code lengths: for every distribution *P* over the dictionary, there exists a prefix code *C* whose length $L_{C}\left(x\right)$ is equal to −logP(x) for all words *x*. Conversely, for every prefix code *C* on the dictionary, there exists a probability measure *P* such that −logP(x) is equal to the code length $L_{C}\left(x\right)$ for all words *x*.

### 2.3. Shannon’s Probability-Based Coding

In his seminal work [1], Shannon proposed the source coding theorem based on a probabilistic framework. Supposing a finite number of words $A_{1}$, $A_{2}$, ⋯, $A_{m}$ are generated from a probabilistic source denoted by a random variable *X* with frequencies $p_{i}$, i=1,⋯,m, then the expected length of any prefix code is no shorter than the entropy of this source defined as $H\left(X\right)=-\sum_{i=1}^{m}\;p_{i}\log p_{i}$. This result offers a lower bound of data compression if a probabilistic model can be assumed. Throughout this paper, we take 2 as the base of the logarithm operation, and thereby bit is the unit of code lengths.

Huffman code is such an optimal code that reaches the expected code length. The codewords are defined by a binary tree constructed from word frequencies. Another well-known method is the Shannon–Fano–Elias code, which uses at most two bits more than the theoretical lower bound. The code length of $A_{i}$ in Shannon–Fano–Elias code is approximately equal to $-\log p_{i}$.

### 2.4. Kolmogorov Complexity and Algorithm-Based Coding

Kolmogorov, who laid the foundation of probability theory, interestingly set aside probabilistic models and, along with other researchers including Solomonoff and Chaitin, pursued an alternative path to understanding the informational structure of data based on the notion of a universal Turing machine. Kolmogorov [10] stated that “information theory must precede probability theory, and not be based on it.”

We give a brief account of some facts about Kolmogorov complexity relevant to our study, and refer readers to Li and Vitányi [11], Vitányi and Li [12] for further detail. A Turing machine is a computer with a finite state operating on a finite symbol set, and is essentially the abstraction of any physical computer that has CPUs, memory, and input and output devices. At each unit of time, the machine reads in one operation command from the program tape, writes some symbols on a work tape, and changes its state according to a transition table. Two important features need more explanation. First, the program is linear, namely, the machine reads the tape from left to right, and never goes back. Second, the program is prefix-free, namely, no program that leads to a halting computation can be the prefix of another such program. This feature is an analog to the concept of prefix-coding. A universal Turing machine can reproduce the results of any other machines. The Kolmogorov complexity of a word *x* with respect to a universal computer U, denoted by $K_{U}\left(x\right)$, is defined as the minimum length overall programs that print *x* and then halt.

The Kolmogorov complexities of all words satisfy the Kraft inequality, due to its natural connection to prefix coding. In fact, for a fixed machine U, we can encode *x* by the minimum length program that prints *x* and halt. Given a long string, if we define a way to parse it into words, we can then encode each word by the above program. Consequently, we encode the string by concatenating the programs one after another. Decoding is straightforward: we input the concatenated program into U, and it reconstructs the original string.

One obvious way of parsing is to take the string itself as the only word. Thus, how much we can compress the string depends on the complexity of this string. At this point, we see the connection between data compression and the Kolmogorov complexity, which is defined for each string with respect to an implementable type of computational machine—the Turing machine.

Next, we highlight some theoretical results about Kolmogorov complexity. First, it is not machine specific except for a machine-specific constant. Second, the Kolmogorov complexity is unfortunately not computable. Third, there exists a universal probability $P_{U}\left(x\right)$ with respect to a universal machine such that $2^{-K(x)}\leq P_{U}\left(x\right)\leq c\;2^{-K(x)}$ for all strings, where *c* is a constant independent of *x*. This means that up to an additive constant, K(x) is equivalent to $-\log P_{U}\left(x\right)$, which can be viewed as the code lengths of a prefix code in light of the Shannon–Fano–Elias code. Because of the non-computability of Kolmogorov complexity, the universal probability is likewise not computable.

The study of the Kolmogorov complexity reveals that the assessment of the exact compression bounds of strings is beyond the ability of any specific Turing machine. However, any program executed on a Turing Machine provides, up to an additive constant, an upper bound on the complexity.

### 2.5. Correspondence Between Probability Models and String Parsing

**Figure 1:**
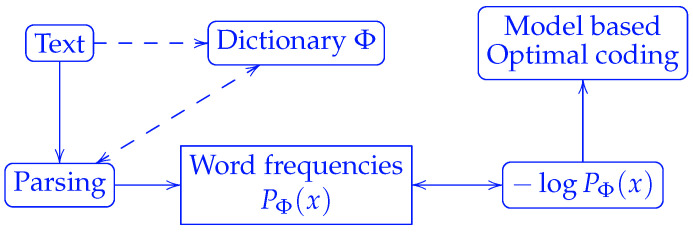
Correspondence between probability models and string parsing by a dictionary. The parsing of a string requires a dictionary Φ, which can be defined either prior to parsing or dynamically during parsing as in Lempel–Ziv coding.

A critical question remaining to be answered in the Shannon source coding theorem is the following: Where does the model that defines probabilities come from? According to the theorem, the optimal code lengths are proportional to the negative logarithm of the word frequencies. Once the dictionary is defined, the word frequencies can be counted for any individual string to be compressed. Equivalently, a dictionary can be induced by the way we parse a string, c.f. Figure 1. It is worth noting that the term “letter” instead of “word” was used in Shannon’s original paper [1], which did not address how to parse strings into words at all.

### 2.6. Fixed-Length and Variable-Length Parsing

The words generated from the parsing process could be either of the same length or of variable lengths. For example, we can encode Shakespeare’s work letter by letter, or encode it by natural words of varying lengths. A choice made at this point leads to two quite different coding schemes.

If we decompose a string into words containing the same number of symbols, this is a fixed-length parsing. The two extra bits for each word is a big deal when each word contains only a few symbols. As the word length gets longer and longer, the relative impact of the two extra bits becomes negligible for each block. An effective alternative to avoid the issue of extra bits is the arithmetic coding, which integrates the codes of successive words at the cost of more computations.

Variable-length parsing decomposes a string into words containing a variable number of symbols. The popular Lempel–Ziv coding is such a scheme. Although the complexity of a string *x* is not computable, the complexity of ‘x1’ relative to ‘*x*’ is small. To concatenate an ‘1’ to the end of ‘*x*’, we can simply use the program that prints *x* followed by printing ‘1’. A recursive implementation of this idea leads to the Lempel–Ziv coding, which concatenates the address of ‘*x*’ and the code of ‘1’.

Please notice that as the data length increases, the dictionary size resulting from the parsing scheme of the Lempel–Ziv coding increases as well—unless an upper limit is imposed. Along the process of encoding, each word occurs only once because, down the road, either it will not be a prefix of any other word, or a new word concatenating it with a certain suffix symbol will be found. To a good approximation, all the words encountered up to a point are equally likely. If we use the same number of bits to store the addresses of these words, their code lengths are equal. Approximately, it obeys Shannon’s source coding theorem too.

### 2.7. Parametric Models and Complexity

Hereafter, we use parametric probabilistic models to count prefix code lengths. The specification of a parametric model includes three aspects: a model class; a model dimension; and parameter values. Suppose we restrict our attention to some hypothetical model classes. Each of these model classes is indexed by a set of parameters, and we define the number of parameters in each model its dimension. We also assume the identifiability of the parameterization, that is, different parameter values correspond to different models. Let us denote one such model class by a probability measure $\left\{P_{\theta }:\theta \in anopenset\Theta \subset {\;R}^{d}\right\}$, and their corresponding frequency functions by {p(x;θ)}. The model class is usually defined by a parsing scheme. For example, if we parse a string symbol by symbol, then the number of words equals the number of symbols appearing in the string. We denote the number of symbols by α, then d=α−1. If we parse the string by every two symbols, then the number of words increases to $d=\alpha^{2}-1$, and so on.

From the above review of Kolmogorov complexity, it is clear that strings themselves do not admit probability models in the first place. Nevertheless, we can fit a string using a parametric model. By doing so, we need to pay extra bits to describe the model as observed by Dr. Rissanen. He termed them as stochastic complexity or parametric complexity. The total code lengths under a model include both the data description and the parametric complexity.

### 2.8. Two References for Code Length Evaluation

The evaluation of redundancy of a given code requires a reference. Two such references are discussed in the literature. In the first scenario, we assume that the words $X^{\left(n\right)}=\left\{X_{1},X_{2}\cdots ,X_{n}\right\}$ are generated according to $P_{\theta_{0}}$ as independent and identically distributed (i.i.d.) random variables, whose outcomes are denoted by $\left\{x_{i}\right\}$. Then the optimal code length is given by $L_{0}=-\sum_{i=1}^{n}\;\log p\left(X_{i};\theta_{0}\right)$. As *n* goes large, its average code length is given by $E\left(L_{0}\right)=nH\left(\theta \right)$. In general, the code length corresponding to any distribution Q(x) is given by $L_{Q}=-\sum_{i=1}^{n}\;\log q\left(X_{i}\right)$, and its redundancy is $R_{Q}=L_{Q}-L_{0}$. The expected redundancy is the Kullback–Leibler divergence between the two distributions:

$$E_{P_{\theta_{0}}}\left(L_{Q}-L_{0}\right)=E_{P_{\theta_{0}}}\log\frac{P_{\theta_{0}}\left(X^{\left(n\right)}\right)}{Q(X^{\left(n\right)})}=D(P_{\theta_{0}}\left||Q\right)\geq 0\;.$$

It can be shown that minmax and maxmin values of average redundancy are equal [13]:

$$\underset{Q}{\inf}\underset{\theta }{\sup}E_{P_{\theta }}\log\frac{P_{\theta }\left(X^{\left(n\right)}\right)}{Q(X^{\left(n\right)})}=\underset{\theta }{\sup}\underset{Q}{\inf}E_{P_{\theta }}\log\frac{P_{\theta }\left(X^{\left(n\right)}\right)}{Q(X^{\left(n\right)})}=I\left(\Theta ;X^{\left(n\right)}\right)\;.$$

A key historical result on redundancy [14,15] is that for each positive number ϵ and for all $\theta_{0}\in \Theta$ except in a set whose volume goes to zero as n⟶∞

(1) $$E_{P_{\theta_{0}}}\left(L_{Q}-L_{0}\right)\geq \frac{d-\epsilon }{2}\log\;n.$$

All these results are about average code length over all possible strings.

Another reference with which any code can be compared is obtained by replacing $\theta_{0}$ by the maximum likelihood estimate ${\hat{\theta }}_{n}$ in $L_{0}$; that is, $L_{{\hat{\theta }}_{n}}=-\sum_{i=1}^{n}\;\log p\left(X_{i};{\hat{\theta }}_{n}\right)$. Please notice that $L_{{\hat{\theta }}_{n}}$ does not satisfy the Kraft inequality. This perspective is a practical one, since in reality $x^{\left(n\right)}$ is simply data without any probability measure. Given a parametric model class $\left\{P_{\theta }\right\}$, we fit the data using one surrogate model that maximizes the likelihood. Then we consider

$$L_{Q}-L_{{\hat{\theta }}_{n}}=\log\frac{p(x^{\left(n\right)};\hat{\theta }\left(x^{\left(n\right)}\right))}{q(x^{\left(n\right)})}\;.$$

### 2.9. Optimality of Normalized Maximum Likelihood Code Length

Minimizing the above quantity leads to the normalized maximum-likelihood (NML) distribution:

$$\hat{p}\left(x^{\left(n\right)}\right)=\frac{p(x^{\left(n\right)};\hat{\theta }\left(x^{\left(n\right)}\right))}{\sum_{x^{\left(n\right)}}\;p\left(x^{\left(n\right)};\hat{\theta }\left(x^{\left(n\right)}\right)\right)}\;.$$

The NML code length is thus given by

(2) $$L_{NML}=-\log\;p\left(x^{\left(n\right)};\hat{\theta }\left(x^{\left(n\right)}\right)\right)+\log\;\sum_{x^{\left(n\right)}}\;p\left(x^{\left(n\right)};\hat{\theta }\left(x^{\left(n\right)}\right)\right)\;.$$

Shtarkov [3] proved the optimality of NML code by showing it solves

(3) $$\min_{q}\max_{x^{\left(n\right)}}\log\frac{p(x^{\left(n\right)};\hat{\theta }\left(x^{\left(n\right)}\right))}{q(x^{\left(n\right)})}\;,$$

where *q* ranges over the set of virtually all distributions. Later Rissanen [2] further proved that NML code solves

$$\min_{q}\max_{g}E_{g}\left[\log\frac{p(X^{\left(n\right)};\hat{\theta }\left(X^{\left(n\right)}\right))}{q(X^{\left(n\right)})}\right]\;,$$

where *q* and *g* range over the set of virtually all distributions. This result states that the NML code is still optimal even if the data are generated from outside the parametric model family. Namely, regardless of the source nature in practice, we can always find the optimal code length from a distribution family.

## 3. Empirical Code Lengths Based on Exponential Family Distributions

In this section, we fit data from a source, either discrete or continuous, by an exponential family due to the following considerations. First, the multinomial distribution, which is used to encode discrete symbols, is an exponential family. Second, according to the Pitman–Koopman–Darmois theorem, exponential families are, under certain regularity conditions, the only models that admit sufficient statistics whose dimensions remain bounded as the sample size grows. On one hand, this property is most desirable in data compression. On the other hand, the results would be valid in the broader context of statistical learning, beyond source coding. Third, as we will show, the first term in the code length expansion is nothing but the empirical entropy for exponential families, which is a straightforward extension of Shannon’s source coding theorem.

### 3.1. Exponential Families

Consider a canonical exponential family of distributions $\left\{P_{\theta }:\theta \in \Theta \right\}$, where the natural parameter space Θ is an open set of $R^{d}$. The density function is given by

(4) $$p\left(x;\theta \right)=\exp\{\theta^{T}\;S\left(x\right)-A\left(\theta \right)\}\;,$$

with respect to some measure μ(dx) on the support of data. The transposition of a matrix (or vector) *V* is represented by $V^{T}$ here and throughout the paper. S(·) is the sufficient statistic for the parameter θ. We denote the first and the second derivatives of A(θ) respectively by $\dot{A}\left(\theta \right)$ and $\ddot{A}\left(\theta \right)$. The entropy or differential entropy of $P_{\theta }$ is $H\left(\theta \right)=A\left(\theta \right)-\theta^{T}\dot{A}\left(\theta \right)$. The following result is an empirical and pathwise version of Shannon’s source coding theorem.

**Theorem** **1.** **[Empirical optimal source code length]** *If we fit an individual data sequence by an exponential family distribution, the NML code length is given by*

(5) $$L_{NML}=nH\left({\hat{\theta }}_{n}\right)+\frac{d}{2}\log\;\frac{n}{2\pi }+\log\int_{\Theta }{\left|I\left(\theta \right)\right|}^{1/2}\;d\theta +o\left(1\right)\;,$$

*where $H({\hat{\theta }}_{n})$ is the entropy evaluated at the maximum likelihood estimate (MLE) ${\hat{\theta }}_{n}=\hat{\theta }\left(x^{\left(n\right)}\right)$, and |I(θ)| is the determinant of the Fisher information $I\left(\theta \right)={\left[-E\left(\frac{\partial^{2}logp(X;\theta )}{\partial \theta_{j}\partial \theta_{k}}\right)\right]}_{j,k=1,\cdots ,d}$. The integral in the expression is taken over the parameter space Θ, and is assumed to be finite.*

Importantly, we do not assume that the data are generated from an exponential family. Rather, for any given data sequence, we fit a distribution from a relevant exponential family to describe the data. In this context, the distribution serves purely as a modeling tool, and any appropriate option from the exponential family toolbox may be used.

The first term in (2) is $nA\left({\hat{\theta }}_{n}\right)-{\left[\sum_{i=1}^{n}S\left(x_{i}\right)\right]}^{T}{\hat{\theta }}_{n}=nA\left({\hat{\theta }}_{n}\right)-n\dot{A}{\left({\hat{\theta }}_{n}\right)}^{T}{\hat{\theta }}_{n}=nH\left({\hat{\theta }}_{n}\right)$, namely, the entropy in Shannon’s theorem except that the model parameter is replaced by the MLE. The second term has a close relationship to the BIC introduced by Akaike [16] and Schwartz [17], and the third term involves the Fisher information which characterizes the local property of a distribution family. Surprisingly and interestingly, this empirical version of the lossless coding theorem brings together three foundational contributions: those of Shannon, Akaike–Schwarz, and Fisher.

Next, we give a heuristic proof of (5) by the local asymptotic normality (LAN) [18], though a complete proof can be found in the Appendix A. In the definition of NML code length (2), the first term becomes empirical entropy for exponential families. Namely,

(6) $$L_{NML}=nH\left({\hat{\theta }}_{n}\right)+\log\;\sum_{x^{\left(n\right)}}\;p\left(x^{\left(n\right)};{\hat{\theta }}_{n}\right)\;.$$

The remaining difficulty is the computation of the summation. In a general problem of data description length, Rissanen [19] derived an analytical expansion requiring five assumptions, which were hard to verify. Here we show for sources from exponential families, the expansion is valid as long as the integral is finite.

Let $U(\theta ,\frac{r}{\sqrt{n}})$ be a cube of size $\frac{r}{\sqrt{n}}$ centering at θ, where *r* is a constant. LAN states that we can expand probability density in each neighborhood $U(\theta ,\frac{r}{\sqrt{n}})$ as follows:

$$\log\frac{p(x^{\left(n\right)};\theta +h)}{p(x^{\left(n\right)};\theta )}=h^{T}\left[\sum_{i=1}^{n}\;S\left(x_{i}\right)-n\dot{A}\left(\theta \right)\right]-\frac{1}{2}h^{T}\left[nI\left(\theta \right)\right]h+o\left(h\right)\;,$$

where $I\left(\theta \right)=\ddot{A}\left(\theta \right)$. Maximizing the likelihood in $U(\theta ,\frac{r}{\sqrt{n}})$ with respect to *h* leads to

$$\max_{h}\log\frac{p(x^{\left(n\right)};\theta +h)}{p(x^{\left(n\right)};\theta )}=\frac{1}{2}{\left[\sum_{i=1}^{n}\;S\left(x_{i}\right)-n\dot{A}\left(\theta \right)\right]}^{T}{\left[nI\left(\theta \right)\right]}^{-1}\left[\sum_{i=1}^{n}\;S\left(x_{i}\right)-n\dot{A}\left(\theta \right)\right]+o\left(\frac{r}{\sqrt{n}}\right)\;.$$

Consequently, if ${\hat{\theta }}_{n}\left(x^{\left(n\right)}\right)$ falls into the neighborhood $U(\theta ,\frac{r}{\sqrt{n}})$, we have

(7) $$p\left(x^{\left(n\right)};{\hat{\theta }}_{n}\right)=e^{\left\{\frac{1}{2}{\left[\sum_{i=1}^{n}\;S\left(x_{i}\right)-n\dot{A}\left(\theta \right)\right]}^{T}{\left[nI\left(\theta \right)\right]}^{-1}\left[\sum_{i=1}^{n}\;S\left(x_{i}\right)-n\dot{A}\left(\theta \right)\right]+o\left(\frac{r}{\sqrt{n}}\right)\right\}}p\left(x^{\left(n\right)};\theta \right)\;,$$

where ${\hat{\theta }}_{n}$ solves $\sum_{i=1}^{n}\;S\left(x_{i}\right)=n\dot{A}\left({\hat{\theta }}_{n}\right)$. Applying the Taylor expansion, we get

$$\sum_{i=1}^{n}\;S\left(x_{i}\right)-n\dot{A}\left(\theta \right)=n\dot{A}\left({\hat{\theta }}_{n}\right)-n\dot{A}\left(\theta \right)=\left[n\ddot{A}\left(\theta \right)\right]\left({\hat{\theta }}_{n}-\theta \right)+o\left(\frac{r}{\sqrt{n}}\right)\;.$$

Plugging it into (7) leads to

(8) $$p\left(x^{\left(n\right)};{\hat{\theta }}_{n}\right)=e^{\left\{\frac{1}{2}{\left({\hat{\theta }}_{n}-\theta \right)}^{T}\left[n\ddot{A}\left(\theta \right)\right]\left({\hat{\theta }}_{n}-\theta \right)+o\left(\frac{r}{\sqrt{n}}\right)\right\}}p\left(x^{\left(n\right)};\theta \right)\;.$$

If we consider i.i.d. random variables $Y_{1},\cdots ,Y_{n}$ sampled from the exponential distribution (4), then the MLE $\hat{\theta }\left(Y^{\left(n\right)}\right)$ is a random variable. The summation of the quantity (8) in the neighborhood $U(\theta ,\frac{r}{\sqrt{n}})$ can be expressed as the following expectation of $\hat{\theta }\left(Y^{\left(n\right)}\right)$:

(9) $$E[e^{\left\{\frac{1}{2}{\left({\hat{\theta }}_{n}-\theta \right)}^{T}\left[n\ddot{A}\left(\theta \right)\right]\left({\hat{\theta }}_{n}-\theta \right)\right\}}1\left({\hat{\theta }}_{n}\in U\left(\theta ,\frac{r}{\sqrt{n}}\right)\right)]\;.$$

Due to the asymptotic normality of MLE $\hat{\theta }\left(Y^{\left(n\right)}\right)$, namely, ${\hat{\theta }}_{n}-\theta \overset{d}{⟶}N\left(0,{\left[nI\left(\theta \right)\right]}^{-1}\right)$, the density of $\hat{\theta }\left(Y^{\left(n\right)}\right)$ is approximated by

$$\frac{{\left|nI\left(\theta \right)\right|}^{1/2}}{{\left(2\pi \right)}^{d/2}}\;e^{\left\{-\frac{1}{2}{\left({\hat{\theta }}_{n}-\theta \right)}^{T}\left[n\ddot{A}\left(\theta \right)\right]\left({\hat{\theta }}_{n}-\theta \right)\right\}}\;d{\hat{\theta }}_{n}\;.$$

Applying this density to the expectation in (9), we find the two exponential terms cancel out, and obtain $\frac{{\left|nI\left(\theta \right)\right|}^{1/2}}{{\left(2\pi \right)}^{d/2}}$. The sum of its logarithm over all neighborhoods $U(\theta ,\frac{r}{\sqrt{n}})$ leads to the remaining terms in (5).

**Figure 2:**
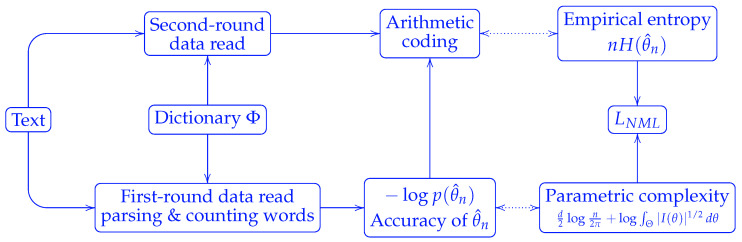
An implementation of NML code length. The data were read in two rounds, one for counting word frequencies, and the other for encoding.

The optimality of the NML code is established in the minimax settings. Yet its implementation requires two passes over the data: one for word counting with respect to a dictionary, and another for encoding, c.f. Figure 2.

### 3.2. Bayesian Predictive Coding

It is natural to ask whether there exists a scheme that passes through the data only once and still can compress the data equally well. It turns out that predictive coding is such a scheme for a given dictionary. The idea of predictive coding is to sequentially make inferences about the parameters in the probability function p(x;θ), which is then used to update the code book. That is, after obtaining observations $x_{1},\cdots ,x_{i}$, we calculate the MLE ${\hat{\theta }}_{i}$, and in turn encode the next observation according to the current estimated distribution. Its code length is thus $L_{predictive}=\left(-\sum_{i=1}^{n}\;\log p\left(X_{i+1}|{\hat{\theta }}_{i}\right)\right)$. This procedure (Rissanen [15,20]) is closely related to the prequential approach to statistical inference as advocated by Dawid [21,22]. Predictive coding is intuitively optimal due to two important fundamental results. First, the MLE ${\hat{\theta }}_{i}$ is asymptotically most accurate since it gathers all the information in $X_{1},\cdots ,X_{i}$ for inference in the parametric model p(x;θ). Second, the code length $\log p(X_{i+1}|{\hat{\theta }}_{i})$ is optimal as dictated by the Shannon source coding theorem. In the case of exponential families, Proposition 2.2 in [5] showed that $L_{predictive}$ can be expanded as follows:

$$L_{predictive}=nH\left({\hat{\theta }}_{n}\right)+\frac{d}{2}\log\;n+{\tilde{D}}_{n}\left(\omega \right)\;,$$

where the sequence of random variables $\left\{{\tilde{D}}_{n}\left(\omega \right)\right\}$ converges to an almost surely finite random variable $\tilde{D}\left(\omega \right)$.

Alternatively, we can use Bayesian estimates in the predictive coding. Starting from a prior distribution w(θ), we encode $x_{1}$ by the marginal distribution $q_{1}\left(x_{1}\right)=\int_{\Theta }p\left(x_{1}|\theta \right)w\left(\theta \right)d\theta$ resulted from w(·). The posterior is given by

$$w_{1}\left(\theta \right)=p\left(x_{1}|\theta \right)w\left(\theta \right)/\int_{\Theta }p\left(x_{1}|\theta \right)w\left(\theta \right)d\theta \;.$$

We then use this posterior as the updated prior to encode the next word $x_{2}$. Using induction, we can show that the marginal distribution to encode the *k*-th word is

$$q_{k}\left(x_{k}\right)=\frac{\int_{\Theta }\left[\prod_{i=1}^{k}\;p\left(x_{i}|\theta \right)\right]w\left(\theta \right)d\theta }{\int_{\Theta }\left[\prod_{i=1}^{k-1}\;p\left(x_{i}|\theta \right)\right]w\left(\theta \right)d\theta }\;.$$

Meanwhile, the updated posterior, also the prior for the next round encoding, becomes

$$w_{k}\left(\theta \right)=\frac{\left[\prod_{i=1}^{k}\;p\left(x_{i}|\theta \right)\right]w\left(\theta \right)}{\int_{\Theta }\left[\prod_{i=1}^{k}\;p\left(x_{i}|\theta \right)\right]w\left(\theta \right)d\theta }\;.$$

**Proposition** **1.** **[Bayesian predictive code length]** *The total Bayesian predictive code length for a string of n words is*

$$L_{B-predictive}=L_{mixture}=-\sum_{k=1}^{n}\;\log q_{k}\left(x_{k}\right)=-\log\int_{\Theta }\left[\prod_{i=1}^{n}\;p\left(x_{i}|\theta \right)\right]w\left(\theta \right)d\theta \;.$$

**Figure 3:**
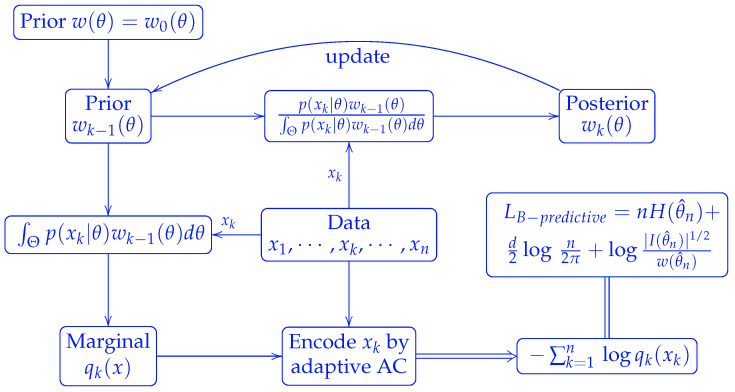
Scheme of Bayesian predictive coding.

Thus the Bayesian predictive code is nothing but the mixture code referred to in reference [4]. The above scheme is illustrated in Figure 3.

**Theorem** **2.** **[Expansion of Bayesian predictive code length]** *If we fit a data sequence by an exponential family distribution, the mixture code length has the expansion*

(10) $$L_{B-predictive}=nH\left({\hat{\theta }}_{n}\right)+\frac{d}{2}\log\;\frac{n}{2\pi }+\log\frac{|I\left({\hat{\theta }}_{n}\right){|}^{1/2}}{w({\hat{\theta }}_{n})}+o\left(1\right)\;,$$

*where w(θ) is any mixture of conjugate prior distributions.*

Once again, we do not assume that the data are generated from an exponential family. Rather than using a single distribution from an exponential family, Bayesian predictive coding employs a mixture of such distributions, thereby generally enhancing its approximation capability.

The result holds for general priors that can be approximated by a mixture of conjugate ones. In the case of multinomial distributions, the conjugate prior is the Dirichlet distribution. Any prior w(θ) continuous on the *d*-dimensional simplex within the cube ${\left[0,1\right]}^{\left(m+1\right)}$ can be uniformly approximated by the Bernstein polynomials of *m* variables, each term of which corresponds to a Dirichlet distribution [23,24]. It is important to note that in the current setting, the source is not assumed to be i.i.d. samples from an exponential family distribution as in Theorem 2.2 and Proposition 2.3 in [5].

When $\int_{\Theta }{\left|I\left(\theta \right)\right|}^{1/2}\;d\theta$ is finite, we can take the Jeffreys prior, $w\left(\theta \right)=\frac{{\left|I\left(\theta \right)\right|}^{1/2}}{{\int |I\left(\theta \right)|}^{1/2}\;d\theta }$ then (10) becomes (5). Putting them together, we have shown that the optimal code length can be achieved by the Bayesian predictive coding scheme.

### 3.3. Redundancy

Now we examine the empirical code length under Shannon’s setting. That is, we evaluate the redundancy of the code length assuming the source is from a hypothetical distribution. The reference is the optimal code length $L_{0}=-\sum_{i=1}^{n}\;\log p\left(X_{i};\theta_{0}\right)$ as introduced in Section 2.8, and $E\left(L_{0}\right)=nH\left(\theta \right)$.

**Proposition** **2.** *If we assume that a source follows an exponential family distribution, then*

(11) $$nH\left({\hat{\theta }}_{n}\right)-L_{0}=-C_{n}\;\log\log\;n+o\left(1\right)\;,$$

*where the sequence of non-negative random variables $\left\{C_{n}\right\}$ have a bounded upper limit, ${\bar{\lim}}_{n\to \infty }\;C_{n}\leq d$, almost surely. If we further assume that $\int_{\Theta }{\left|I\left(\theta \right)\right|}^{1/2}\;d\theta <\infty$, then*

(12) $$R_{NML}=L_{NML}-L_{0}=\frac{d}{2}\log\;\frac{n}{2\pi }-C_{n}\;\log\log\;n+\log\int_{\Theta }{\left|I\left(\theta \right)\right|}^{1/2}\;d\theta +o\left(1\right)\;,$$

*where $\left\{C_{n}\right\}$ is the same as the above.*

The left side of (11) is $\left[-\sum_{i=1}^{n}\;\log p\left(X_{i}|{\hat{\theta }}_{n}\right)\right]-\left[-\sum_{i=1}^{n}\;\log p\left(X_{i}|\theta_{0}\right)\right]$, and the rest is true according to the proof of Proposition 2.2 in [5], Equation (18). The NML code is a special case of the mixture code, whose redundancy is given by Theorem 2.2 in [5]. The details on the law of the iterated logarithm can be found in the book [25]. We note that ${\bar{\lim}}_{n\to \infty }\;C_{n}$ is bounded below by 1. This proposition confirms that $nH({\hat{\theta }}_{n})$ is an underestimate of the theoretical optimal code length. Notably, this setting in which the data is assumed to originate from a probabilistic source of fixed dimension serves only the theoretical analysis.

### 3.4. Coding of Discrete Symbols and Multinomial Model

For compressing strings of discrete symbols, it is sufficient to consider the discrete distribution specified by a probability vector, $\theta =(p_{1},p_{2},\cdots ,p_{d},p_{d+1})$, where $\sum_{k=1}^{d+1}\;p_{k}=1$. Its frequency function is $P\left(X=k\right)=\prod_{k=1}^{d+1}\;p_{k}^{1(X=k)}$. The Fisher information matrix $I(p_{1},\;\cdots \;p_{d})$ can be shown to be

$$-{\left[E\frac{\partial^{2}logP(X=k)}{\partial p_{j}\partial p_{k}}\right]}_{j,k=1,\cdots ,d}=\left(\begin{array}{cccc} \frac{1}{p_{1}}+\frac{1}{p_{d+1}} & \frac{1}{p_{d+1}} & \cdots & \frac{1}{p_{d+1}}\\ \frac{1}{p_{d+1}} & \frac{1}{p_{2}}+\frac{1}{p_{d+1}} & \cdots & \frac{1}{p_{d+1}}\\ \vdots & \vdots & \ddots & \vdots \\ \frac{1}{p_{d+1}} & \frac{1}{p_{d+1}} & \cdots & \frac{1}{p_{d}}+\frac{1}{p_{d+1}} \end{array}\right)\;.$$

Thus $|I\left(p_{1},\;\cdots \;p_{d}\right)|=1/\prod_{k=1}^{d+1}p_{k}$.

Suppose $X_{1},\cdots ,X_{n}$ are i.i.d. random variables obeying the above discrete distribution. Then $S=\sum_{i=1}^{n}\;X_{i}$ follows a multinomial distribution $Multi(n;p_{1},p_{2},\cdots ,p_{d},p_{d+1})$. Its conjugate prior distribution is the Dirichlet distribution $\left(\alpha_{1},\alpha_{2},\cdots ,\alpha_{d+1}\right)$, whose density function is

$$\frac{\Gamma (\sum_{k=1}^{d+1}\alpha_{k})}{\prod_{k=1}^{d+1}\Gamma \left(\alpha_{k}\right)}\prod_{k=1}^{d+1}\;p_{k}^{\alpha_{k}-1}\;,$$

where $\Gamma \left(t\right)=\int_{u=0}^{+\infty }u^{-t}e^{-u}du$. Since the Jeffreys prior is proportional to $|I\left(p_{1},\;\cdots \;p_{d}\right){|}^{1/2}$, in this case, it equals Dirichlet(1/2,1/2, ⋯, 1/2), whose density is $\frac{\Gamma ((d+1)/2)}{\Gamma {\left(1/2\right)}^{d+1}}\prod_{k=1}^{d+1}\;p_{k}^{-1/2}$. The Jeffreys prior was also used by Krichevsky [14] to derive optimal universal codes.

It is noticed that $\Gamma \left(1/2\right)=\sqrt{\pi }$. Plug it into Equation (10), we have the following specific form of the NML code length for the multinomial distribution. Remember that the distribution or word frequencies are specific for a given dictionary Φ, and we thus term it as $L_{NML@\Phi }$. If we change the dictionary, the code length changes accordingly.

**Proposition** **3.** **[Optimal code lengths for a multinomial distribution]**

(13) $$L_{NML@\Phi }=nH\left({\hat{\theta }}_{n}\right)+\frac{d}{2}\log\;n-\frac{d}{2}-\log\Gamma \left(\frac{d+1}{2}\right)+\frac{1}{2}\log\pi \;,$$

*where $nH\left({\hat{\theta }}_{n}\right)=-\sum_{k=1}^{d}{\hat{p}}_{k}\log{\hat{p}}_{k}$, ${\hat{p}}_{k}=n_{k}/n$—the frequency of the k-th word appearing in the string.*

## 4. Compression of Random Sequences and DNA Sequences

### 4.1. Lossless Compression Bound and Description Length

Given a dictionary of words, we parse a string into words followed by counting their frequencies ${\hat{p}}_{k}=n_{k}/n$, the total number of words *n*, and the number of distinct words *d*. Plugging them into expression (13), we obtain the lossless compression bound for this dictionary or parsing. If a different parsing is tried, the three quantities—word frequencies, number of words, dictionary size (number of distinct words)—would change, and the resulting bound would change accordingly. In the general situation where the data are not necessarily discrete symbols, we replace the code length with description length (10) as termed by Rissanen.

Since each parsing corresponds to a probabilistic model, the code length is model dependent. The comparison of two or more coding schemes is exactly the selection of models, with the expression (13) as the objective function.

### 4.2. Rissanen’s Principle of Minimum Description Length and Model Selection

Rissanen, in his works [15,19,26], proposed the principle of minimum description length (MDL) as a more general modeling rule than that of maximum likelihood, which was recommended, analyzed, and popularized by R. A. Fisher. From the information-theoretic point of view, when we encode data from a source by prefix coding, the optimal code is the one that achieves the minimum description length. Because of the equivalence between a prefix code length and the negative logarithm of the corresponding probability distribution—via Kraft’s inequality –this leads naturally to the modeling principle: the MDL principle. That is, one should choose the model or prefix coding algorithm that gives the minimal description of data; see Hansen and Yu [27] for a review on this topic. We also refer readers to [6,28] for a more complete account of the MDL principle.

MDL is a mathematical formulation of the general principle known as Occam’s razor: choose the simplest explanation consistent with the observed data [7]. We make one remark about the significance of MDL. On the one hand, Shannon’s work establishes the connection between optimal coding and probabilistic models. On the other hand, Kolmogorov’s algorithmic theory says that the complexity, or the absolute optimal coding, cannot be proved by any Turing machines. MDL offers a practical principle: it allows us to make choices among possible models and coding algorithms without requiring proof of optimality. As more model candidates are evaluated over time, our understanding continues to progress.

### 4.3. Compression Bounds of Random Sequences

A random sequence is incompressible by any model-based or algorithmic prefix coding as indicated by the complexity results [11,12]. Thus a legitimate compression bound of a random sequence should be no less than one up to certain variations. Conversely, if the compression rates of a sequence using $L_{NML@\Phi }$ as the compression bound are no less than 1 under all dictionaries Φ, namely,

$$\min_{\Phi :dictionaries}\;\frac{L_{NML@\Phi }}{L_{RAW}}=1+\frac{L_{NML@\Phi }-L_{RAW}}{L_{RAW}}\geq 1\;,$$

where $L_{RAW}$ is the theoretical bit-length of the raw sequence, then the sequence can be considered random. If we assume the source follows a uniform distribution, $L_{RAW}=nH$ while $L_{NML@\Phi }$ can be calculated by (13). Although testing all dictionaries is challenging, we can experiment with a subset—particularly those suggested by the domain experience. More theoretical analysis on randomness testing based on universal codes can be found in the book [29].

### 4.4. A Simulation Study: Compression Bounds of Pseudo-Random Sequences

Simulations were carried out to test the theoretical bounds. First, a pseudo-random binary string of size 3000 was simulated in R according to Bernoulli trials with a probability of 0.5. In Table 1, the first column shows the word length used for parsing the data; The second column shows the word number; and the third column shows the number of distinct words. We group the terms in (13) into three parts: the term involving *n*, the term involving logn, and others. The bounds by $nH({\hat{\theta }}_{n})$, $nH\left({\hat{\theta }}_{n}\right)+\frac{d}{2}\log\;n$ and $L_{NML}$ in (13) are respectively shown in the next three columns. As the word length increases, *d* increases, and the bounds by $nH({\hat{\theta }}_{n})$ exhibit a decreasing trend. A bound smaller than 1 indicates the sequence can be compressed, contradicting the assertion that random sequences cannot be so. When the word length is 8, the dictionary size is 375, and the bound by $nH({\hat{\theta }}_{n})$ is only 0.929. The incompressibility nature of random sequences falsifies $nH({\hat{\theta }}_{n})$ as a legitimate compression bound. If the logn term is included, the bounds are always larger than 1. The bounds by $L_{NML}$ (13) are tighter while remaining larger than 1—except the case at the bottom row, where the number of distinct words approaches the total number of words, making the expansion insufficient. Since $L_{NML}$ is an achievable bound, $nH\left({\hat{\theta }}_{n}\right)+\frac{d}{2}\log\;n$ is an overestimate.

### 4.5. Knowledge Discovery by Data Compression

On the other hand, if we can compress a sequence by a certain prefix coding scheme, then this sequence is not random. In the meantime, this coding scheme presents a clue to understanding the information structure hidden in the sequence. Data compression is one general learning mechanism, among others, to discover knowledge from nature and other sources.

Ryabko, Astola, and Gammerman [30] applied the idea of Kolmogorov complexity to the statistical testing of some typical hypotheses. This approach was used to analyze DNA sequences in [31].

### 4.6. DNA Sequences of Proteins

The information carried by the DNA double helix consists of two long complementary strings of the letters A, G, C, and T. It is interesting to see if we can compress DNA sequences at all. Next, we carried out the lossless compression experiments on a couple of protein-encoding DNA sequences.

### 4.7. Rediscovery of the Codon Structure

In Table 2, we present the result of applying the NML code length $L_{NML}$ in (13) to an *E. coli* protein gene sequence labeled by *b0059* [32], which has 2907 nucleotides. Each row corresponds to one model used for encoding. All the models being tested are listed in the first column. In the first model, we encode the DNA nucleotides one by one and name it Model 1. In the second or third model, we parse the DNA sequence by pairs and then encode the resulting dinucleotide sequence according to their frequencies. Different starting positions result two different phases, denoted by 2.0 and 2.1, respectively. Other models are understood in the same fashion. Note that all these models are generated by fixed-length parsing. The last model “a.a.” means we first translate DNA triplets into amino acids and then encode the resulting amino acid sequence. The second column shows the total number of words in each parsed sequence. The third column shows the number of different words in each parsed sequence, or the dictionary size. The fourth column is the empirical entropy estimated from observed frequencies. The next column is the first term in expression (13), which is the product of the second and fourth columns. Then we calculate the rest terms in (13). The total bits are then calculated, and the compression rates are the ratios $L_{NML}/\left(2907*2\right)$. The last column displays the compression rates for each model.

All the compression rates are around 1, except for the rate obtained from Model 3.0, which aligns with the correct codon and phase. Thus the comparison of compression bounds rediscovers the codon structure of this protein-coding DNA sequence and the phase of the open reading frame. It is somewhat surprising that the optimal code length $L_{NML}$ allows us to mathematically identify the triplet coding system using only the sequence of one gene. Historically, this system was discovered by Francis Crick and his colleagues in the early 1960s using frame-shift mutations on bacteria-phage T4.

Next, we take a closer look at the results. The compression rate of the four-nucleotide word coding is closest to 1, and thus it behaves more like “random”. For example, it is 0.9947 for Model 4.2. The first term of empirical entropy contributes 5431 bits, while the rest terms contribute 346 bits. If we use $\frac{d}{2}\log\;n$ instead, the rest term is 0.5∗(219−1)∗log(726)≈1036 bits, and the compression rate becomes 1.11, which is less tight. If the Ziv–Lempel algorithm is applied to the *b0059* sequence, 635 words are generated along the way. Each word requires log(635) bits for keeping the address of its prefix and 2 bits for the last nucleotide. In total, it needs 635∗log(635)=5912 bits for storing addresses, and 635∗2=1270 bits for storing the words’ last symbol. The compression rate of Ziv–Lempel coding is 1.24.

### 4.8. Redundant Information in Protein Gene Sequences

It is known that the $4^{3}=64$ triplets correspond to only 20 amino acids plus stop codons. Thus redundancy does exist in protein-coding sequences. Most of the redundancy lies in the third position of a codon. For example, GGA, GGC, GGT, and GGG all correspond to glycine. According to Table 2, the amino acid sequence contains 4048.22 bits of information, while there are 5277.51 bits of information in Model 3.0. Thus the redundancy in this sequence is estimated to be (5277.51 − 4048.22)/4048.22 = 0.30.

### 4.9. Randomization

To evaluate the accuracy or significance of the compression rates of a DNA sequence, we need a reference distribution for comparison. A common method is to consider the randomness obtained by permutations. That is, given a DNA sequence, we permute the nucleotide bases and re-calculate the compression rates. By repeating this permutation procedure, we generate a reference distribution.

In Table 3, we examine the compression rates for *E. coli* ORF *b0060*, which has 2352 nucleotides. First, the optimal compression rate of 0.958 is achieved with model 3.0. Second, we further carry out the calculations for permuted sequences. The averages, standard deviations, and 1% (or 99%) quantiles of compression rates under different models are shown in Table 3 as well. Except for Model 1, all the compression rates, in terms of either averages or 1% quantiles are above 1 for both $L_{NML}$ and $nH\left({\hat{\theta }}_{n}\right)+\frac{d}{2}\log\;n$. Third, the results by the single term $nH({\hat{\theta }}_{n})$ are about 0.996, 0.994, 0.986, and 0.952, respectively, for one-, two-, three-, and four-nucleotide models. The 99% quantiles of $nH({\hat{\theta }}_{n})$ for the four-nucleotide models are no larger than 0.961. Fourth, the results of $nH\left({\hat{\theta }}_{n}\right)+\frac{d}{2}\log\;n$ show extra bits compared to those of $L_{NML}$, and the compression ratio go from 1.02 to 1.17, suggesting the rest terms in (13) are not negligible.

It is noted Models 3.1 and 3.2 are obtained by phase-shifting from the correct Model 3.0. Other models are obtained by incorrect parsing. These models can serve as references for Model 3.0. The incorrect parsing and phase-shifting resemble the behavior of the linear congruential pseudo-random number generator, and serve as a form of randomization.

## 5. Discussion

Putting together the analytical results and numerical examples, we show the compression bound of a data sequence using an exponential family is the code length derived from the NML distribution (5). The empirical bound can be implemented by the Bayesian predictive coding for any given dictionary or model. Different models are then compared by their empirical compression bounds.

The examples of DNA sequences and pseudo-random sequences indicate that the compression rates by any dictionary are indeed larger than 1 for random sequences, in line with the assertions of the Kolmogorov complexity theory. Conversely, if significant compression is achieved by a specific model, certain knowledge is gained. The codon structure is such an instance.

Unlike the algorithmic complexity, which includes an additive constant, the results based on probability distributions give the exact bits of code lengths. All three terms in (5) are important for the compression bound. Using only the first term $nH({\hat{\theta }}_{n})$ can lead to bounds of random sequences smaller than 1. The discrepancy increases as the dictionary size grows as seen from Table 1 and Table 3. The bound by adding the second term $\frac{d}{2}\log\;n$ had been proposed by the two-part coding or the Kolmogorov complexity. It is equivalent to BIC widely used in model selection. However, it overestimates the influence of the dictionary size as shown by the examples of simulations and DNA sequences. The inclusion of the Fisher information in the third term results in a tighter bound. The terms other than $nH({\hat{\theta }}_{n})$ become larger as the dictionary size increases in Table 1 and Table 3. The observation that the compression bounds—considering all terms in (5)—remain slightly above 1 for all tested libraries meets our expectation on the incompressibility of random sequences.

Although the empirical compression bound is obtained under the i.i.d. model, the word length can be set sufficiently large to capture the local dependencies between symbols. Indeed, as shown in the examples of DNA sequences, the empirical entropy term in (5) may become smaller, for either the original sequences or the permuted ones. Meanwhile, the second term become larger. For a specific sequence, a better dictionary is selected by trading off the entropy term against the model complexity term.

Rissanen [19] obtained an expansion of the NML code length, in which the first term is the log-likelihood of data with the parameters plugged in by the MLE. In this article, we show it is exactly the empirical entropy if the parametric model takes any exponential family. According to this formulation, the NML code length can be viewed as an empirical and pathwise version of Shannon’s source coding theorem. Furthermore, the asymptotics in [19] requires five assumptions, which are hard to examine. Suzuki and Yamanishi proposed a Fourier approach to calculate the NML code length [33] for continuous random variables with certain assumptions. In contrast, we show that (5) is valid for exponential families, as long as $\int_{\Theta }{\left|I\left(\theta \right)\right|}^{1/2}\;d\theta <\infty$, without any additional assumptions. If the Jeffreys prior is improper in the interior of the full parameter space, we can restrict the parameter to a compact subset. Exponential families include not only distributions of discrete symbols, such as the multinomial but also continuous distributions, such as the normal distribution.

The mathematics underlying the expansion of NML is the structure of local asymptotic normality as proposed by LeCam [18]. LAN has been used to demonstrate the optimality of certain statistical estimates. This article connects LAN to compression bound. We have shown as long as LAN is valid, a similar expansion to (2) can be obtained.

Compared to Shannon’s source coding theorem that assumes the data are from a source with a given distribution, we reported empirical versions that apply to each individual data sequence without any a priori distributional assumption. In contrast to the average optimal code length in Shannon’s theorem, the results presented in Theorems 1 and 2 are sequence specific, although distributions are used as tools for analysis. We describe the data by fitting distributions from exponential families and mixtures of exponential families, which are sufficient for most scenarios in coding and statistical learning. Knowledge discovery is illustrated through an example involving DNA sequences. The results are primarily conceptual, and their connection to recent progress in data compression—such as [34]—is worth further investigation.

## Figures and Tables

**Table 1:** The data compression rates of a binary string of size 3000 under different parsing models. The data were simulated in R according to Bernoulli trials with probability 0.5.

Parsing the String by Nine Libraries			Compression Rates by Three Quantities		
Word Length	n: Word Number	d: Size of Dictionary	$\mathrm{nH}({\hat{\theta }}_{n})$	$\mathrm{nH}({\hat{\theta }}_{n})$ $+\frac{d}{2}\log\;n$	$L_{\mathrm{NML}}$ (13)
1	3000	2	0.999918	1.001843	1.001952
2	1500	4	0.998591	1.003866	1.003641
3	1000	8	0.998961	1.010587	1.008834
4	750	16	0.995422	1.019299	1.012974
5	600	32	0.989603	1.037285	1.018977
6	500	64	0.981597	1.075738	1.027959
7	428	124	0.964885	1.144324	1.031261
8	375	196	0.928930	1.206829	1.006312
9	333	252	0.872958	1.223846	0.950283

**Table 2:** The data compression rates of *E. coli* ORF *b0059* calculated by (13) under different parsing models.

Model	*n*: Word	*d*: Size	Empirical	1st	Rest	$L_{\mathrm{NML}@\Phi }$	Compression
	Number	of Φ	Entropy	Term	Terms	Total Bits	Rate
1	2907	4	1.9924	5792.00	16.58	5808.58	0.9991
2.0	1453	16	3.9570	5749.49	59.81	5809.31	0.9995
2.1	1453	16	3.9425	5728.44	59.81	5788.25	0.9959
**3.0**	**969**	**58**	**5.2842**	**5120.39**	**157.11**	**5277.51**	**0.9077**
3.1	968	63	5.5905	5411.63	167.13	5578.76	0.9605
3.2	968	64	5.6706	5489.10	169.11	5658.21	0.9742
4.0	726	218	7.4507	5409.24	345.07	5754.31	0.9908
4.1	726	217	7.4337	5396.87	344.20	5741.07	0.9885
4.2	726	219	7.4814	5431.49	345.940	5777.43	0.9947
4.3	726	221	7.4678	5421.64	347.67	5769.31	0.9933
a. a.	969	21	4.1056	3978.31	69.92	4048.22	0.6963

**Table 3:** The data compression rates of *E. coli* ORF *b0060* and statistics from permutations. The protein-coding gene sequence consists of 2352 nucleotide bases. The second row shows the parsing models, and the third row shows the compression rates computed by $L_{NML}$ for the raw sequence. The subsequent rows present statistics obtained from permutations, in order, for $L_{NML}$ (13), $nH({\hat{\theta }}_{n})$, and $nH\left({\hat{\theta }}_{n}\right)+\frac{d}{2}\log\;n$, respectively. For $nH({\hat{\theta }}_{n})$, the 99%-quantiles are shown, all of which are smaller than 1. For the other two, the 1%-quantiles are shown, all which are larger than 1 except in Model 1.0.

		Models Generated by Different Parsing									
		1.0	2.0	2.1	3.0	3.1	3.2	4.0	4.1	4.2	4.3
$L_{NML}$ (13)	raw data	0.999	1.000	1.001	0.958	0.980	0.989	1.001	1.002	0.997	0.999
bound definition	statistics of permutations										
$L_{NML}$ (13)	average	0.999	1.006	1.006	1.020	1.020	1.020	1.020	1.020	1.020	1.020
	SD ($\times 10^{-3}$)	0.00	0.74	0.76	1.69	1.77	1.71	4.22	4.33	4.15	3.92
	1%-quantile	0.999	1.004	1.004	1.016	1.016	1.016	1.011	1.010	1.011	1.011
$nH({\hat{\theta }}_{n})$	average	0.996	0.994	0.994	0.986	0.987	0.986	0.952	0.952	0.952	0.952
	SD ($\times 10^{-3}$)	0.00	0.74	0.76	1.69	1.77	1.71	3.69	3.79	3.63	3.42
	99%-quantile	0.996	0.995	0.995	0.990	0.990	0.990	0.961	0.961	0.960	0.960
$nH\left({\hat{\theta }}_{n}\right)+\frac{d}{2}\log\;n$	average	0.999	1.010	1.010	1.051	1.051	1.051	1.174	1.174	1.174	1.174
	SD ($\times 10^{-3}$)	0.00	0.74	0.76	1.70	1.78	1.71	7.49	7.66	7.37	7.01
	1%-quantile	0.999	1.008	1.008	1.046	1.047	1.047	1.157	1.156	1.157	1.157

## Data Availability

The numerical results reported in the article can be reproduced by the Supplementary Files. One file with R code corresponds to Table 1. The other with Python 3.13 code corresponds to Table 2 and Table 3.

## Supplementary Materials

The following supporting information can be downloaded at: https://www.mdpi.com/article/10.3390/e27080864/s1.

## Abbreviations

The following abbreviations are used in this manuscript:

NML	normalized maximum likelihood
$L_{NML@\Phi }$	NML code length under dictionary Φ
$L_{B-predictive}$	Bayesian predictive code length
AC	arithmetic coding
LAN	local asymptotic normality

## Appendix A

This section contains the proofs of the results in Section 2 and Section 3. The following basic facts about the exponential family (4) are needed, see [35].

1. $E\left(S\left(X\right)\right)=\dot{A}\left(\theta \right)$, and $Var\left(S\left(X\right)\right)=\ddot{A}\left(\theta \right)$.
2. $\dot{A}\left(\cdot \right)$ is one to one on the natural parameter space.
3. The MLE ${\hat{\theta }}_{n}$ based on $\left(X_{1},\cdots ,X_{n}\right)$ is given by ${\hat{\theta }}_{n}={\dot{A}}^{-1}\left({\bar{S}}_{n}\right)$, where ${\bar{S}}_{n}=\frac{1}{n}\sum_{i=1}^{n}\;S\left(X_{i}\right)$.
4. The Fisher information matrix $I\left(\theta \right)=\ddot{A}\left(\theta \right)$.

**Proof** **of Theorem 1.** In the canonical exponential family, the natural parameter space is open and convex. Since $\int_{\Theta }{\left|I\left(\theta \right)\right|}^{1/2}\;d\theta <\infty$, we can find a series of bounded set $\left\{\Theta^{\left(k\right)},k=1,2,\cdots \right\}$ such that $\log[\int_{\Theta }{\left|I\left(\theta \right)\right|}^{1/2}\;d\theta ]-\log[\int_{\Theta^{\left(k\right)}}{\left|I\left(\theta \right)\right|}^{1/2}\;d\theta ]=\epsilon_{k}$. where $\epsilon_{k}\to 0$. Furthermore, we can select each bounded set $\Theta^{\left(k\right)}$ so that it can be partitioned into disjoint cubes, each of which is denoted by $U(\theta_{j}^{\left(k\right)},\frac{r}{\sqrt{n}})$ with $\theta_{j}^{\left(k\right)}$ as its center and $\frac{r}{\sqrt{n}}$ as its side length. Namely, $\Theta^{\left(k\right)}={⋃}_{j}U\left(\theta_{j}^{\left(k\right)},\frac{r}{\sqrt{n}}\right)$, and $U\left(\theta_{j_{1}}^{\left(k\right)},\frac{r}{\sqrt{n}}\right)⋂U\left(\theta_{j_{2}}^{\left(k\right)},\frac{r}{\sqrt{n}}\right)=\emptyset$ for $j_{1}\neq j_{2}$. The normalizing constant in Equation (6) can be summed (integration in the case of continuous variables) by the sufficient statistic $\sum_{i=1}^{n}S\left(x_{i}\right)$, and in turn by the MLE ${\hat{\theta }}_{n}$

(A1) $$\sum_{\left\{x^{\left(n\right)},\;{\hat{\theta }}_{n}\in \Theta^{\left(k\right)}\right\}}\;p\left(x^{\left(n\right)};{\hat{\theta }}_{n}\right)=\sum_{U(\theta_{j}^{\left(k\right)},\frac{r}{\sqrt{n}})}\sum_{\left\{x^{\left(n\right)},\;{\hat{\theta }}_{n}\in U\left(\theta_{j}^{\left(k\right)},\frac{r}{\sqrt{n}}\right)\right\}}\;p\left(x^{\left(n\right)};{\hat{\theta }}_{n}\right)\;,$$

(A2) $$p\left(x^{\left(n\right)};{\hat{\theta }}_{n}\right)=e^{\left\{{\hat{\theta }}_{n}^{T}\sum_{i=1}^{n}\;S\left(x_{i}\right)-nA\left({\hat{\theta }}_{n}\right)\right\}}\mu^{n}\left(dx^{n}\right)=e^{\left\{n{\hat{\theta }}_{n}^{T}{\bar{S}}_{n}-nA\left({\hat{\theta }}_{n}\right)\right\}}\mu^{n}\left(dx^{n}\right)\;.$$

Now expand $n[\theta {\bar{S}}_{n}-A\left(\theta \right)]$ around ${\hat{\theta }}_{n}$ within the neighborhood $U(\theta_{j}^{\left(k\right)})$.

$$\begin{array}{ccc} n[\theta^{T}{\bar{S}}_{n}-A\left(\theta \right)] & = & n\left[{\hat{\theta }}_{n}^{T}{\bar{S}}_{n}-A\left({\hat{\theta }}_{n}\right)\right]+{\left(\theta -{\hat{\theta }}_{n}\right)}^{T}\left[n{\bar{S}}_{n}-n\dot{A}\left({\hat{\theta }}_{n}\right)\right]\\ & & -\frac{1}{2}{\left(\theta -{\hat{\theta }}_{n}\right)}^{T}\left[n\ddot{A}\left({\hat{\theta }}_{n}\right)\right]\left(\theta -{\hat{\theta }}_{n}\right)+M_{1}n||\theta -{\hat{\theta }}_{n}{\left|\right|}^{3}\;. \end{array}$$

Since the MLE ${\hat{\theta }}_{n}={\dot{A}}^{-1}\left({\bar{S}}_{n}\right)$, the second term is zero. Furthermore, we expand $\ddot{A}\left({\hat{\theta }}_{n}\right)$ around $\ddot{A}\left(\theta \right)$, and rearrange the terms in the equation, then we have

$$n\left[{\hat{\theta }}_{n}^{T}{\bar{S}}_{n}-A\left({\hat{\theta }}_{n}\right)\right]=n\left[\theta^{T}{\bar{S}}_{n}-A\left(\theta \right)\right]+\frac{1}{2}{\left({\hat{\theta }}_{n}-\theta \right)}^{T}\left[n\ddot{A}\left(\theta \right)\right]\left({\hat{\theta }}_{n}-\theta \right)+M_{2}n||\theta -{\hat{\theta }}_{n}{\left|\right|}^{3}\;,$$

where the constant $M_{2}$ involves the third-order derivatives of A(θ), which is continuous in the canonical exponential family and thus bounded in the bounded set $\Theta^{\left(k\right)}$. In other words, $M_{2}$ is bounded uniformly across all $\left\{U\left(\theta_{j}^{\left(k\right)},\frac{r}{\sqrt{n}}\right)\right\}$. Similar bounded constants will be used repeatedly hereafter. Then Equation (A2) becomes

$$p\left(x^{\left(n\right)};{\hat{\theta }}_{n}\right)=e^{\frac{1}{2}{\left(\theta -{\hat{\theta }}_{n}\right)}^{T}\left[n\ddot{A}\left(\theta \right)\right]\left(\theta -{\hat{\theta }}_{n}\right)+M_{2}n||\theta -{\hat{\theta }}_{n}{\left|\right|}^{3}}e^{n[\theta {\bar{S}}_{n}-A\left(\theta \right)]}\mu^{n}\left(dx^{n}\right)\;.$$

Notice that the exponential form $e^{n[\theta {\bar{S}}_{n}-A\left(\theta \right)]}$ is the density of $n\bar{S}$. If we consider i.i.d. random variables $Y_{1},\cdots ,Y_{n}$ sampled from the exponential distribution (4), the MLE $\hat{\theta }\left(Y^{\left(n\right)}\right)$ is a random variable. Take $\theta =\theta_{j}^{\left(k\right)}$, then the sum of the above quantity over the neighborhood $U(\theta_{j}^{\left(k\right)},\frac{r}{\sqrt{n}})$ is nothing but the expectation of $\hat{\theta }\left(Y^{\left(n\right)}\right)={\dot{A}}^{-1}\left({\bar{S}}_{n}\right)$ with respect to the distribution of $n{\bar{S}}_{n}$, evaluated at the parameter $\theta_{j}^{\left(k\right)}$:

$$\begin{array}{ccc} & & \sum_{\left\{x^{\left(n\right)},\;{\hat{\theta }}_{n}\in U\left(\theta_{j}^{\left(k\right)},\frac{r}{\sqrt{n}}\right)\right\}}\;p\left(x^{\left(n\right)};{\hat{\theta }}_{n}\right)\\ & = & E_{\theta_{j}^{\left(k\right)}}\left[1\left[{\hat{\theta }}_{n}\in U\left(\theta_{j}^{\left(k\right)},\frac{r}{\sqrt{n}}\right)\right]e^{\frac{1}{2}{\left({\hat{\theta }}_{n}-\theta_{j}^{\left(k\right)}\right)}^{T}\left[n\ddot{A}\left(\theta_{j}^{\left(k\right)}\right)\right]\left({\hat{\theta }}_{n}-\theta_{j}^{\left(k\right)}\right)+M_{2}n||{\hat{\theta }}_{n}-\theta_{j}^{\left(k\right)}{\left|\right|}^{3}}\right]\;. \end{array}$$

Let $\xi_{n}=\sqrt{n}\left({\hat{\theta }}_{n}-\theta_{j}^{\left(k\right)}\right)$. Now ${\hat{\theta }}_{n}\in U(\theta_{j}^{\left(k\right)},\frac{r}{\sqrt{n}}$) if and only if $\xi_{n}\in U\left(0,r\right)$, where U(0,r) is the d-dimensional cube centered at zero with the side length *r*. Next expanding $e^{M_{2}n||{\hat{\theta }}_{n}-\theta_{j}^{\left(k\right)}{\left|\right|}^{3}}$ in the neighborhood, the above becomes

$$\sum_{\left\{{\hat{\theta }}_{n}\in U\left(\theta_{j}^{\left(k\right)},\frac{r}{\sqrt{n}}\right)\right\}}\;p\left(x^{\left(n\right)};{\hat{\theta }}_{n}\right)=E[1\left[\xi_{n}\in U\left(0,r\right)\right]\left[e^{\frac{1}{2}\xi_{n}^{T}I\left(\theta_{j}^{\left(k\right)}\right)\xi_{n}}\left(1+M_{3}n^{-\frac{1}{2}}\right)\right]\;.$$

According to the central limit theorem, $n^{-\frac{d}{2}}\left[\sum_{i=1}^{n}\;S\left(Y_{i}\right)-\dot{A}\left(\theta_{j}^{\left(k\right)}\right)\right]\overset{d}{⟶}N\left(0,\ddot{A}\left(\theta_{j}^{\left(k\right)}\right)\right)$. Moreover, the approximation error has the Berry–Esseen bound $O(n^{-\frac{1}{2}})$, where the constant is determined by the bound on A(θ)’s third-order derivatives. Similarly, we have the asymptotic normality of MLE, $\xi_{n}\left(Y^{\left(n\right)}\right)\overset{d}{⟶}N\left(0,I{\left(\theta_{j}^{\left(k\right)}\right)}^{-1}\right)$, where the Berry–Esseen bound is valid for the convergence, see [36]. Therefore, the expectation converges as follows:

$$\begin{array}{ccc} & & E\{1\left[\xi_{n}\in U\left(0,r\right)\right]e^{\frac{1}{2}\xi_{n}^{T}I\left(\theta_{j}^{\left(k\right)}\right)\xi_{n}}\left(1+M_{3}n^{-\frac{1}{2}}\right)\}\\ & = & \int \;1\left[\xi_{n}\in U\left(0,r\right)\right]\left[e^{\frac{1}{2}\xi_{n}^{T}I\left(\theta_{j}^{\left(k\right)}\right)\xi_{n}}\left(1+M_{3}n^{-\frac{1}{2}}\right)\right]\frac{|I\left(\theta_{j}^{\left(k\right)}\right){|}^{1/2}}{{\left(2\pi \right)}^{d/2}}\;e^{-\frac{1}{2}\xi_{n}^{T}I\left(\theta_{j}^{\left(k\right)}\right)\xi_{n}}\;d\xi_{n}+M_{4}n^{-\frac{1}{2}}\\ & = & {\left(2\pi \right)}^{-\frac{d}{2}}|I\left(\theta_{j}^{\left(k\right)}\right){|}^{\frac{1}{2}}\int \;1\left[\xi_{n}\in U\left(0,r\right)\right]\left(1+M_{3}n^{-\frac{1}{2}}\right)]d\xi_{n}+M_{4}n^{-\frac{1}{2}}\\ & = & {\left(2\pi \right)}^{-\frac{d}{2}}{\left|I\left(\theta_{j}^{\left(k\right)}\right)\right|}^{\frac{1}{2}}r^{d}\left(1+M_{3}n^{-\frac{1}{2}}\right)+M_{4}n^{-\frac{1}{2}}\\ & = & n^{\frac{d}{2}}{\left(2\pi \right)}^{-\frac{d}{2}}{\left|I\left(\theta_{j}^{\left(k\right)}\right)\right|}^{1/2}\left(r^{d}n^{-\frac{d}{2}}\right)\left(1+M_{3}^{\prime }n^{-\frac{1}{2}}\right)\;. \end{array}$$

Plugging this into the sum (A1), we obtain

$$\begin{array}{ccc} & & \sum_{\left\{x^{\left(n\right)},\;{\hat{\theta }}_{n}\in \Theta^{\left(k\right)}\right\}}\;p\left(x^{\left(n\right)};{\hat{\theta }}_{n}\right)=n^{\frac{d}{2}}{\left(2\pi \right)}^{-\frac{d}{2}}[\sum_{U(\theta_{j}^{\left(k\right)},\frac{r}{\sqrt{n}})}\;|I\left(\theta_{j}^{\left(k\right)}\right){|}^{1/2}\left(r^{d}n^{-\frac{d}{2}}\right)]\left(1+M_{3}^{\prime }n^{-\frac{1}{2}}\right)\\ & ⟶ & n^{\frac{d}{2}}{\left(2\pi \right)}^{-\frac{d}{2}}[\int_{\Theta^{\left(k\right)}}{\left|I\left(\theta \right)\right|}^{1/2}\;d\theta ]\left(1+M_{3}^{\prime }n^{-\frac{1}{2}}\right)]\; \end{array}$$

$$\begin{array}{ccc} & & \log\left[\sum_{\left\{x^{\left(n\right)},\;{\hat{\theta }}_{n}\in \Theta^{\left(k\right)}\right\}}\;p\left(x^{\left(n\right)};{\hat{\theta }}_{n}\right)\right]=\frac{d}{2}\log\;\frac{n}{2\pi }+\log\int_{\Theta^{\left(k\right)}}{\left|I\left(\theta \right)\right|}^{1/2}\;d\theta +M_{3}^{\prime \prime }n^{-\frac{1}{2}}\\ & = & \frac{d}{2}\log\;\frac{n}{2\pi }+\log\int_{\Theta }{\left|I\left(\theta \right)\right|}^{1/2}\;d\theta +[\log\int_{\Theta^{\left(k\right)}}{\left|I\left(\theta \right)\right|}^{1/2}\;d\theta -\log\int_{\Theta }{\left|I\left(\theta \right)\right|}^{1/2}\;d\theta ]\\ & & +M_{3}^{\prime \prime }n^{-\frac{1}{2}}\\ & = & \frac{d}{2}\log\;\frac{n}{2\pi }+\log\int_{\Theta }{\left|I\left(\theta \right)\right|}^{1/2}\;d\theta -\epsilon_{k}+M_{3}^{\prime \prime }n^{-\frac{1}{2}}\;. \end{array}$$

Note that the bound $M_{3}^{\prime \prime }$ of the last term relies solely on $\Theta^{\left(k\right)}$. For a given *k*, we select *n* such that the last term is sufficiently small. This completes the proof. □

**Proof** **of Theorem 2.** First we consider the conjugate prior of (4), which takes the form

(A3) $$u\left(\theta \right)=\exp\{\alpha^{\prime }\;\theta \;-\beta \;A\left(\theta \right)-B\left(\alpha ,\beta \right)\}\;,$$

where α is a vector in $R^{d}$, β is a scalar, and $B\left(\alpha ,\beta \right)=\log\;\int_{\Theta }\exp\left\{\alpha^{\prime }\;\theta \;-\beta \;A\left(\theta \right)\right\}\;d\theta$. Then the marginal density is

(A4) $$m\left(x^{\left(n\right)}\right)=\exp\left\{B\left(\sum_{i=1}^{n}T\left(x_{i}\right)+\alpha ,n+\beta \right)-B\left(\alpha ,\beta \right)\right\}\;,$$

according to the definition of B(·,·). Therefore

$$L_{mixture}=B\left(\alpha ,\beta \right)-B\left(\sum_{i=1}^{n}S\left(x_{i}\right)+\alpha ,n+\beta \right)=B\left(\alpha ,\beta \right)-\log(\int_{\Theta }\exp\left\{nL_{n}\left(t\right\}\;d\;t\right)\;,$$

where $nL_{n}\left(t\right)={\left[\sum_{i=1}^{n}S\left(x_{i}\right)+\alpha \right]}^{T}t-\left(n+\beta \right)A\left(t\right).$ The minimum of $L_{n}\left(t\right)$ is achieved at

$${\tilde{\theta }}_{n}=\tilde{\theta }\left(x^{\left(n\right)}\right)={\dot{A}}^{-1}\left(\frac{\sum_{i=1}^{n}S\left(x_{i}\right)+\alpha }{n+\beta }\right)\;.$$

Notice that

$$\dot{A}\left({\tilde{\theta }}_{n}\right)=\frac{\sum_{i=1}^{n}S\left(x_{i}\right)+\alpha }{n+\beta }=\frac{\sum_{i=1}^{n}S\left(x_{i}\right)}{n}+O\left(\frac{1}{n}\right)=\dot{A}\left({\hat{\theta }}_{n}\right)+O\left(\frac{1}{n}\right)\;.$$

Through Taylor’s expansion, it can be shown that

(A5) $${\tilde{\theta }}_{n}={\hat{\theta }}_{n}+O\left(\frac{1}{n}\right)\;.$$

Notice that $-{\ddot{L}}_{n}\left(t\right)=\frac{n+\beta }{n}\ddot{A}\left(t\right)\;.$ Let $\Sigma =-{\ddot{L}}_{n}^{-1}\left({\tilde{\theta }}_{n}\right)=\frac{n}{n+\beta }{\ddot{A}}^{-1}\left({\tilde{\theta }}_{n}\right)\;.$ By expanding $L_{n}\left(t\right)$ at the saddle point ${\tilde{\theta }}_{n}$ and applying the Laplace method (see [37]), we have

$$\begin{array}{ccc} \log(\int_{\Theta }\exp\left\{nL_{n}\left(t\right)\right\}\;d\;t) & = & -\frac{d}{2}\log\;\frac{n}{2\pi }+\frac{1}{2}\log\left(det\;\Sigma \right)+nL_{n}\left({\tilde{\theta }}_{n}\right)+O\left(\frac{1}{n}\right)\\ & ⟶ & -\frac{d}{2}\log\;\frac{n}{2\pi }-\frac{1}{2}\log\left(det\;I\left({\tilde{\theta }}_{n}\right)\right)+nL_{n}\left({\tilde{\theta }}_{n}\right)\;. \end{array}$$

Next,

$$\begin{array}{ccc} & & L_{mixture}-nH\left({\hat{\theta }}_{n}\right)\\ & ⟶ & B\left(\alpha ,\beta \right)+\frac{d}{2}\log\;\frac{n}{2\pi }+\frac{1}{2}\log\left(det\;I\left({\tilde{\theta }}_{n}\right)\right)-nL_{n}\left({\tilde{\theta }}_{n}\right)-nH\left({\hat{\theta }}_{n}\right)\\ & = & B\left(\alpha ,\beta \right)+\frac{d}{2}\log\;\frac{n}{2\pi }+\frac{1}{2}\log\left(det\;I\left({\tilde{\theta }}_{n}\right)\right)\\ & & -{\left[\sum_{i=1}^{n}S\left(x_{i}\right)+\alpha \right]}^{T}{\tilde{\theta }}_{n}+\left(n+\beta \right)A\left({\tilde{\theta }}_{n}\right)-nA\left({\hat{\theta }}_{n}\right)+{\left[\sum_{i=1}^{n}S\left(x_{i}\right)\right]}^{T}{\hat{\theta }}_{n}\\ & = & \frac{d}{2}\log\;\frac{n}{2\pi }+\frac{1}{2}\log\left(det\;I\left({\tilde{\theta }}_{n}\right)\right)-\left[\alpha^{T}{\tilde{\theta }}_{n}-\beta A\left({\tilde{\theta }}_{n}\right)-B\left(\alpha ,\beta \right)\right]\\ & & -{\left[\sum_{i=1}^{n}S\left(x_{i}\right)\right]}^{T}\left(\tilde{\theta }-{\hat{\theta }}_{n}\right)+n\left[A\left({\tilde{\theta }}_{n}\right)-A\left({\hat{\theta }}_{n}\right)\right]\\ & = & \frac{d}{2}\log\;\frac{n}{2\pi }+\frac{1}{2}\log\left(det\;I\left({\tilde{\theta }}_{n}\right)\right)-\log w\left({\tilde{\theta }}_{n}\right)\\ & & -\left[\sum_{i=1}^{n}S{\left(x_{i}\right)}^{T}\left(\tilde{\theta }-{\hat{\theta }}_{n}\right)+n\dot{A}{\left(\hat{\theta }\right)}^{T}\left({\tilde{\theta }}_{n}-{\hat{\theta }}_{n}\right)+\frac{n}{2}{\left({\tilde{\theta }}_{n}-{\hat{\theta }}_{n}\right)}^{T}\ddot{A}\left({\hat{\theta }}_{n}\right)\left({\tilde{\theta }}_{n}-{\hat{\theta }}_{n}\right)\right]+O\left(\frac{1}{n}\right)\\ & = & \frac{d}{2}\log\;\frac{n}{2\pi }+\frac{1}{2}\log\left(det\;I\left({\tilde{\theta }}_{n}\right)\right)-\log w\left({\tilde{\theta }}_{n}\right)\\ & & -n\left[{\left[{\bar{S}}_{n}-\dot{A}\left({\hat{\theta }}_{n}\right)\right]}^{T}\left({\tilde{\theta }}_{n}-{\hat{\theta }}_{n}\right)+\frac{1}{2}{\left({\tilde{\theta }}_{n}-{\hat{\theta }}_{n}\right)}^{T}\ddot{A}\left({\hat{\theta }}_{n}\right)\left({\tilde{\theta }}_{n}-{\hat{\theta }}_{n}\right)\right]+O\left(\frac{1}{n}\right)\\ & ⟶ & \frac{d}{2}\log\;\frac{n}{2\pi }+\frac{1}{2}\log\left(det\;I\left({\hat{\theta }}_{n}\right)\right)-\log w\left({\hat{\theta }}_{n}\right)\;. \end{array}$$

The last step is valid because of ${\hat{\theta }}_{n}={\dot{A}}^{-1}\left({\bar{S}}_{n}\right)$ and (A5). This proves the case of the prior u(θ) in (A3). Meanwhile, we obtain the expansion of (A4)

(A6) $$\begin{array}{ccc} & & m\left(x^{\left(n\right)}\right)=\exp\left\{-L_{mixture}\right\}=\exp\left\{B\left(\sum_{i=1}^{n}S\left(x_{i}\right)+\alpha ,n+\beta \right)-B\left(\alpha ,\beta \right)\right\}\\ & = & \exp\{-nH\left({\hat{\theta }}_{n}\right)-\frac{d}{2}\log\;\frac{n}{2\pi }-\frac{1}{2}\log\left(det\;I\left({\hat{\theta }}_{n}\right)\right)+\log w\left({\hat{\theta }}_{n}\right)+o\left(1\right)\}\\ & = & \left[w\left({\hat{\theta }}_{n}\right)+o\left(1\right)\right]\exp\left\{-nH\left({\hat{\theta }}_{n}\right)-\frac{d}{2}\log\;\frac{n}{2\pi }-\frac{1}{2}\log\left(det\;I\left({\hat{\theta }}_{n}\right)\right)\right\}\;. \end{array}$$

If the prior of θ takes the form of a finite mixture of the conjugate distributions (A3) as in the following

(A7) $$w\left(\theta \right)=\sum_{j=1}^{J}\lambda_{j}\;\exp\left\{\alpha_{j}^{\prime }\;\theta \;-\beta_{j}\;A\left(\theta \right)-B\left(\alpha_{j},\beta_{j}\right)\right\}=\sum_{j=1}^{J}\lambda_{j}\;u_{j}\left(\theta \right)\;,$$

where $\sum_{j=1}^{J}\lambda_{j}=1$, $0<\lambda_{j}<1,j=1,\cdots ,J$. Then the marginal density is given by

$$\begin{array}{ccc} & & m\left(x^{\left(n\right)}\right)=\sum_{j=1}^{J}\lambda_{j}\;\exp\left\{B\left(\sum_{i=1}^{n}T\left(x_{i}\right)+\alpha_{j},n+\beta_{j}\right)-B\left(\alpha_{j},\beta_{j}\right)\right\}\\ & = & \sum_{j=1}^{J}\lambda_{j}\left[u_{j}\left({\hat{\theta }}_{n}\right)+o\left(1\right)\right]\exp\left\{-nH\left({\hat{\theta }}_{n}\right)-\frac{d}{2}\log\;\frac{n}{2\pi }-\frac{1}{2}\log\left(det\;I\left(\hat{\theta }\right)\right)\right\}\\ & = & \exp\left\{-nH\left({\hat{\theta }}_{n}\right)-\frac{d}{2}\log\;\frac{n}{2\pi }-\frac{1}{2}\log\left(det\;I\left({\hat{\theta }}_{n}\right)\right)\right\}\left[\sum_{j=1}^{J}\lambda_{j}u_{j}\left({\hat{\theta }}_{n}\right)+o\left(1\right)\right]\\ & = & exp\left\{-nH\left({\hat{\theta }}_{n}\right)-\frac{d}{2}\log\;\frac{n}{2\pi }-\frac{1}{2}\log\left(det\;I\left({\hat{\theta }}_{n}\right)\right)\right\}\left[w\left({\hat{\theta }}_{n}\right)+o\left(1\right)\right]\\ & = & \exp\{-nH\left({\hat{\theta }}_{n}\right)-\frac{d}{2}\log\;\frac{n}{2\pi }-\frac{1}{2}\log\left(det\;I\left({\hat{\theta }}_{n}\right)\right)+\log w\left({\hat{\theta }}_{n}\right)+o\left(1\right)\}\;. \end{array}$$

Each summand is approximated by (A6). This completes the proof because of $L_{mixture}=-\log\left\{m\left(x^{\left(n\right)}\right)\right\}$. □
